# Assessment of depression symptoms among cancer patients: a cross-sectional study from a developing country

**DOI:** 10.1038/s41598-024-62935-x

**Published:** 2024-05-24

**Authors:** Maher Battat, Nawal Omair, Mohammad A. WildAli, Aidah Alkaissi, Riad Amer, Amer A. Koni, Husam T. Salameh, Sa’ed H. Zyoud

**Affiliations:** 1 Bone Marrow Transplant and Leukemia Unit, An-Najah National University Hospital, Nablus, 44839 Palestine; 2 Department of Hematology and Oncology, An-Najah National University Hospital, Nablus, 44839 Palestine; 3 Department of Nursing, College of Medicine and Health Sciences, An-Najah National University, Nablus, 44839 Palestine; 4 Department of Medicine, College of Medicine and Health Sciences, An-Najah National University, Nablus, 44839 Palestine; 5 Division of Clinical Pharmacy, Department of Hematology and Oncology, An-Najah National University Hospital, Nablus, 44839 Palestine; 6 Department of Clinical and Community Pharmacy, College of Medicine and Health Sciences, An-Najah National University, Nablus, 44839 Palestine; 7 Poison Control and Drug Information Center (PCDIC), College of Medicine and Health Sciences, An-Najah National University, Nablus, 44839 Palestine; 8 Clinical Research Center, An-Najah National University Hospital, Nablus, 44839 Palestine

**Keywords:** Palestine, Depression symptoms, Cancer patients, Oncology, BDI-II, Cancer, Health care, Oncology

## Abstract

Cancer patients experience psychological symptoms such as depression during the cancer treatment period, which increases the burden of symptoms. Depression severity can be assessed using the beck depression inventory (BDI II). The purpose of the study was to use BDI-II scores to measure depression symptoms in cancer patients at a large tertiary hospital in Palestine. A convenience sample of 271 cancer patients was used for a cross-sectional survey. There are descriptions of demographic, clinical, and lifestyle aspects. In addition, the BDI-II is a tool for determining the severity of depression. Two hundred seventy-one patients participated in the survey, for a 95% response rate. Patients ranged in age from 18 to 84 years, with an average age of 47 years. The male-to-female ratio was approximately 1:1, and 59.4% of the patients were outpatients, 153 (56.5%) of whom had hematologic malignancies. Most cancer patients (*n* = 104, 38.4%) had minimal depression, while 22.5%, 22.1%, and 17.0% had mild, moderate, and severe depression, respectively. Education level, economic status, smoking status, and age were significantly associated with depression. The BDI-II is a useful instrument for monitoring depressive symptoms. The findings support the practice of routinely testing cancer patients for depressive symptoms as part of standard care and referring patients who are at a higher risk of developing psychological morbidity to specialists for treatment as needed.

## Introduction

Cancer is the second leading cause of mortality both worldwide and in Palestine, accounting for 14% of all deaths, after heart disease (30%)^1,2^. Additionally, the anticipated rise in cancer diagnoses among Palestinians is likely to place added strain on the already stretched financial and infrastructural resources of the healthcare system, particularly given the prevailing financial and political uncertainties^2–5^. As advancements in cancer treatments progress, more patients are experiencing either complete cures or extended life expectancies. As a result, there is increased focus on the emotional challenges that come with being diagnosed with and treated for cancer. Research indicates that approximately 30% of patients experience mental health issues such as anxiety, depression, and adjustment disorders^6^, although the exact prevalence varies depending on the specific condition^7^. Treating depression can lead to better emotional and psychological health, even amidst the physical toll of cancer symptoms. Mood plays a significant role in how patients perceive their quality of life (QoL) and the extent of their suffering. It can begin at the time of diagnosis and continue beyond the completion of cancer treatment^8^.

Depression is associated with decreased functional status, decreased adherence to treatment, longer hospitalizations, and the desire to die sooner^9^. Almost 25% of cancer patients experience severe depressive symptoms, whereas 77% of those with advanced disease experience severe depressive symptoms^10^. Depression is common in cancer patients and is strongly associated with oropharyngeal (22–57%), pancreatic (33 to 50%), breast (1.5–46%), and lung (11 to 44%) cancer. Patients with other malignancies, such as colon (13–25%), gynecological (12–23%), and lymphoma (8–19%), had a lower frequency of depression^7^.

Depressive symptoms are often identical to those of physical illness or its treatments, making it difficult to diagnose depression in physically ill people. This is especially true when a cancer patient is diagnosed with depression. Many of the symptoms needed to diagnose depression are often caused by cancer treatments (e.g. chemotherapy, biological therapy), such as fatigue, weight loss, anhedonia (the inability to feel pleasure in typically enjoyable activities) and psychomotor retardation.

Depression, also known as clinical depression or severe depressive disorder, is a prevalent yet serious mood disorder. It manifests as severe symptoms that impact a person's emotions, thoughts, and ability to cope with daily activities such as sleeping, eating, and working. These symptoms must persist for at least two weeks to be diagnosed^7^.

Various techniques have been created to evaluate how well symptoms are managed, aiding in the recognition of linked symptoms. For instance, the beck depression inventory (BDI II)-21 is capable of evaluating prevalent psychological symptoms among cancer patients. The BDI-II remains instrumental in exploring the characteristics and evaluation of depression. Its effectiveness as a screening tool in patients with medical conditions and cancer has been examined in multiple research studies, establishing it as a reliable self-reported assessment tool^11^.

Cancer patients suffer many symptoms during cancer progression that negatively affect their QoL. Importantly, there is no comforting care assessment tool, such as the BDI, available for cancer patients in Palestine. Therefore, the purpose of this study was to evaluate cancer patients’ reported depression symptoms using the beck depression scale (BDS) at a large tertiary care hospital. However, to our knowledge, this is the first study assessing depression using the BDI-II in occupied Palestinian territories in the context of mental health.

## Methods

### Study design

The research objectives were pursued through a quantitative cross-sectional investigation.

### Study setting

An-Najah National University Hospital (NNUH) was established in 2013 through a partnership with the Faculty of Medicine and Health Sciences. NNUH stands as Palestine's sole facility, offering sophisticated electrophysiology, intricate open-heart surgeries, autologous bone marrow transplants, and specialized care for both adult and pediatric leukemia patients. NNUH encompasses various medical units, an emergency ward, dialysis facilities, radiology services, and ultrasound and tomography departments and has a capacity of 120 beds^12^.

### Study population

Cancer patients receive comprehensive care through outpatient oncology clinics and inpatient services at NNUH. These services cover various procedures, including diagnosis, chemotherapy, autologous bone marrow transplantation (autoBMT), and the management of treatment side effects or complications. In certain oncological scenarios, such as neutropenic fever, patients may require immediate attention, leading to visits to the emergency room followed by hospital admission. This integrated approach ensures that patients receive comprehensive and timely care for their cancer treatment needs at NNUH.

### Sample size

During the research period spanning from April 2021 to August 2021, the NNUH received an average of 600 cancer patients each month. This number served as the basis for determining the necessary sample size for analysis. Using the Raosoft sample size calculator with a response distribution of 0.50, an error margin of 5%, and a confidence interval of 95%, a preliminary sample size of 235 was calculated. However, to accommodate potential dropout rates, this figure was adjusted, indicating a requirement of 259 patients. To bolster the study's robustness and mitigate the risk of erroneous results, an additional 10% of the sample (24 patients) was included, bringing the final targeted sample size to 285. The validity and reliability of the questionnaire were evaluated through content validity, construct validity, and reliability testing methods. Hence, we only used triangulation, involving two hemato-oncology physicians, three oncology nurses, and one statistician, to verify the validity of the data. We also assessed the consistency of 11 patients (22 questionnaires) between their two visits. Moreover, after creating the questionnaire, we piloted it on 11 patients, making adjustments as necessary based on their feedback.

### Sampling procedure

The researchers utilized convenience sampling, involving 271 cancer patients.

### Inclusion and exclusion criteria

#### Inclusion criteria

- Ensuring that patients agree to participate is vital for ethical research.
- The ability to read and write at age 18 ensures that patients can understand the study information and provide feedback.
- Specifying cancer and hematologic malignancies keeps the study focused on the relevant patient population. Both inpatients and outpatients are included to capture a broader range of experiences.

#### Exclusion criteria

- Patients in the ICU may be too critically ill to participate effectively.
- Patients in a coma were unable to consent or participate in the study.
- Patients with preexisting cognitive issues may not be able to understand or participate in the study reliably.
- Isolated patients may have difficulty communicating or require special protocols that the study may not be equipped for.

### Data collection instrument

Patients completed the questionnaires themselves, and nurses explained the questions if patients requested further clarification. All surveys were performed on paper and then analyzed using an electronic database. This study involved a secondary analysis of previously published data using various approaches in another study on factors related to palliative care symptoms in cancer patients in Palestine^13^. The data included patient demographics and clinical characteristics collected at various points during cancer treatment, such as diagnosis, chemotherapy, clinic visits, AutoBMT, advanced cancer stages, outpatient and inpatient oncology visits, and related factors. The data were collected over 5 months, from April to August 2021. Patients were provided with the Arabic version of the BDI-II^14^ by either the researcher or a designated nurse and were encouraged to fill it out themselves, with assistance available if needed.

The surveys were kept in a designated location within particular departments designed for adult patients with cancer. These departments included outpatient oncology clinics, medical oncology units, vascular units, surgical units, bone marrow transplant and leukemia units, and surgical cardiac care units. Furthermore, the researcher gathered additional medical data from the patients' records. Approximately 15 patients chose not to participate, and 10 surveys were unfinished. Assessment tools for psychological symptoms, such as the BDI-II, provide a baseline assessment and evaluation for depression in cancer patients.

### Beck depression inventory (BDI) II

Many factors contribute to the variation in depression incidence, including patient age and sex, medical status, cancer diagnosis, and cancer stage^15^. Hence, these inquiries also aid in evaluating depression among individuals with cancer. Moreover, questions regarding the diagnostic approach (such as inclusion or substitution methods), the type of assessment utilized (including diagnostic interviews or self-reported measures), and the criteria for inclusion (whether clinical or subclinical) are crucial for assessing depression within this demographic group.

The BDI, short for Beck Depression Inventory, is a tool consisting of 21-point self-assessment ratings designed to gauge attitudes and symptoms of depression. Completing the BDI typically takes approximately 10 min, yet individuals are required to possess a reading level equivalent to fifth or sixth grade to comprehend the questionnaire adequately. Clinicians employ this inventory to ascertain the severity of depression in individuals and tailor appropriate therapeutic interventions. The BDI was developed by Aaron T. Beck, a prominent psychiatrist recognized as the pioneer of cognitive behavior therapy^16^.

Depression is a medical condition characterized by a prolonged feeling of sadness. This leads to a lack of interest in previously enjoyable activities and can greatly disrupt daily life. While experiencing sadness is normal in response to events such as the loss of a loved one, financial strain, relationship issues, or job loss, clinical depression occurs when these feelings persist for an extended period without an obvious cause^16^.

### Questions on the BDI-II

The BDI-II comprises 21 inquiries aligned with the diagnostic criteria outlined in the DSM-V, which professionals use to assess mental health conditions. Each question offers multiple-choice responses with scores ranging from 0 to 3. These questions address various aspects, such as feelings of sadness, pessimism, past failures, loss of pleasure, guilt, self-criticism, suicidal thoughts, agitation, changes in sleeping and eating patterns, concentration difficulties, fatigue, and diminished interest in activities once enjoyed.

### Scores on the BDI-II

The Beck Depression Inventory (BDI) utilizes a straightforward scoring method in which each of the four multiple-choice options is given a score ranging from 0 to 3. After all 21 questions are answered, the total points are tallied. Based on the total score, the severity of depression was categorized as follows: no depression (0–13 points), mild depression (14–19 points), moderate depression (20–28 points), or severe depression (29–63 points)^16^.

### Statistical analysis

The data were analyzed using the Social Sciences Statistical Package (SPSS) version 21. Basic demographic data were summarized using descriptive statistics. When comparing continuous variables provided as the median and interquartile range, Mann‒Whitney *U*/Kruskal‒Wallis tests were used. P values less than 0.05 were considered to indicate statistical significance.

### Ethics approval and consent to participate

The Institutional Review Board (IRB) of An-Najah National University and the NNUH administrator approved this study. All methods used in the study were conducted in accordance with relevant guidelines and regulations, including the Helsinki Declaration. Participants signed an informed consent form guaranteeing data privacy, and all the data were kept confidential and used exclusively for research purposes.

## Results

### Demographic data

The study included 271 participants, for a response rate of 95%. Table 1 shows the distribution of cancer types among our sample. Among them, 52% were younger than 50 years, and the majority (67.9%, *n* = 184) were married. The average age was 47.17 years, ranging from 18 to 84 years. The gender distribution was nearly equal, with 51.3% men and 48.7% women. In terms of education, the majority (67.5%, *n* = 183) had completed high school, while 32.5% (n = 88) had completed university or college. Regarding socioeconomic status, 53.9% had a low income (< 2000 NIS), 38.4% had a middle income (2000–5000 NIS), and only 7.7% had a high income (> 5000 NIS).

**Table 1:** Cancer types.

Cancer types	Frequency (%)
Hematologic malignancies	
Acute lymphoblastic leukemia	26 (9.1%)
Chronic lymphocytic leukemia	6 (2.1%)
Acute myeloid leukemia	28 (9.8%)
Hodgkin lymphoma	32 (11.2%)
Non-Hodgkin lymphoma	33 (11.5%)
Multiple myeloma	26 (9.1%)
Myelodysplastic syndrome	4 (1.4%)
Solid tumors	
Breast	37 (12.9%)
Colon	15 (5.2%)
Rectal	8 (2.8%)
Sigmoid	3 (1%)
Gastric	8 (2.8%)
Duodenum	2 (0.7%)
Pelvic retroperetonial mass	3 (1%)
Sarcoma	9 (3.1%)
Uterine	5 (1.7%)
Ovarian	7 (2.4%)
Teratoma	1 (0.3%)
Bladder	5 (1.7%)
Pancreatic	10 (3.5%)
Gallbladder	2 (0.7%)
Lung	6 (2.1%)
Hepatiocellular carcinoma	1 (0.3%)
Nasopharyngeal	1 (0.3%)
Vocal cord	1 (0.3%)
Larynx	1 (0.3%)
Prostate	3 (0.3%)
Malignant misothelioma	1 (0.3%)
Esophageal	1 (0.3%)

Among all participants, 22.1% were smokers, 4.8% had deformities such as Tal Hashomer syndrome, 36.5% were employed, 47.6% lived in villages, and 41.0% resided in cities. Furthermore, 59.4% were outpatients, and 56.5% were diagnosed with hematologic malignancies. Notably, the majority of cancer patients (88.9%, *n* = 241) were receiving treatment, with 75.6% actively receiving chemotherapy. Family psychological support was the most common form of support (59.8%), followed by support from healthcare teams (44.3%), religious support (38.0%), and social support (34.3%) (see Table 2).

**Table 2:** Association between patient characteristics and depression (BDI-II).

Variable	Frequency (%)	Depression, median [Q1-Q3]	*P* value
Age			**0.024**
≤ 50	141 (52.0)	15.0 [9.0–24.0]	
> 50	130 (48.0)	18.5 [11.0–25.0]	
Gender			0.815
Male	139 (51.3)	17.0 [10.0–26.0]	
Female	132 (48.7)	16.0 [10.3–24.0]	
Marital status			0.226
Single	87 (32.1)	15.0 [9.0–25.0]	
Married	184 (67.9)	17.0 [10.3–24.0]	
Educational level			** < 0.001**
School	183 (67.5)	18.0 [11.0–27.0]	
University or collage	88 (32.5)	14.0 [7.0–20.0]	
Socioeconomic status			** < 0.001**
Low-income	146 (53.9)	18.5 [11.8–27.3]	
Middle-income	104 (38.4)	15.0 [9.3–21.8]	
High-income	21 (7.7)	10.0 [4.5–15.5]	
Deformities			0.571
Yes	13 (4.8)	17.0 [11.5–27.0]	
No	258 (95.2)	16.0 [10.0–24.0]	
Smoker			**0.004**
Yes	60 (22.1)	20.0 [13.0–29.0]	
No	211 (77.9)	16.0 [10.0–24.0]	
Work			0.074
Yes	99 (36.5)	16.0 [8.0–22.0]	
No	172 (63.5)	17.0 [11.0–27.0]	
Living location			0.751
Urban	111 (41.0)	17.0 [10.0–25.0]	
Rural	129 (47.6)	16.0 [10.0–24.0]	
Camp or refugee	31 (11.4)	16.0 [10.0–24.0]	
Hospitalization status			0.234
Inpatient	110 (40.6)	17.0 [11.0–26.0]	
Outpatient	161 (59.4)	16.0 [10.0–24.0]	
Type of cancer			0.066
Hematology	153 (56.5)	16.0 [9.0–24.0]	
Solid	118 (43.5)	17.5 [11.0–26.3]	
Treatment stage			0.647
Yes	241 (88.9)	16.0 [10.0–24.0]	
No	30 (11.1)	17.5 [10.8–27.3]	
Currently on chemotherapy			0.466
Yes	205 (75.6)	16.0 [10.0–24.0]	
No	66 (24.4)	17.5 [11.7–26.3]	
Recently, pancytopenia			0.588
Yes	86 (31.7)	17.0 [10.0–25.3]	
No	185 (68.3)	16.0 [10.0–24.0]	
AutoBMT			0.547
Yes	24 (8.9)	16.5 [10.0–27.7]	
No	247 (91.1)	17.0 [10.0–24.0]	
Admitted for surgery			0.688
Yes	10 (3.7)	17.0 [11.5–28.3]	
No	261 (96.3)	17.0 [10.0–24.0]	
Types of psychological support			0.264
Family support	162 (59.8)	Yes 16.0 [10.8–24.0]	
		No 17.0 [10.0–27.0]	
Social support	93 (34.3)	Yes 17.0 [10.5–24.0]	0.885
		No 16.5 [10.0–25.0]	
Religious support	103 (38.0)	Yes 16.0 [11.0–24.0]	0.839
		No 17.0 [10.0–25.0]	
Health care team support	120 (44.3)	Yes 17.0 [11.0–25.8]	0.168
		No 16.0 [9.0–24.0]	

Significant values are in bold.

### The severity among cancer patients according to the BDI-II

The majority of cancer patients (38.4%) had minimal depression, while 22.5%, 22.1%, and 17.0% had mild, moderate, or severe depression, respectively (see Table 3). However, the median BDI score [Q1-Q3] was 17.0 [10.0–24.0], and the mean ± SD was 18.2 ± 11.0.

**Table 3:** BDI-II categories.

Depression severity		Frequency	Percent
Valid	0–13 (minimal)	104	38.4
	14–19 (mild)	61	22.5
	20–28 (moderate)	60	22.1
	29–63 (severe)	46	17
	Total	271	100

The associations between patient characteristics and depression are shown in Table 2. The results showed that cancer patients over 50 years of age had significantly more depression than those younger than 50 years of age did (*p* = 0.024), and the median BDI score was 18.5 [11.0–25.0] for the > 50 years age group and 15.0 [9.0–24.0] for the > 50 years age group. A significant difference was also found in the categories of educational level (*p* < 0.001), where cancer patients with low educational levels had higher depression scores than those with higher educational levels (university or college). Furthermore, poor socioeconomic status was significantly associated with increased depression intensity. The current study showed that smokers had moderate depression, as indicated by a BDI-II score of 20.0 [13.0–29.0], while nonsmokers had mild depression, with a BDI-II score of 16.0 [10.0–24.0]. This difference was significant *(p* = 0.004). Other factors, such as sex, social status, type of cancer, hospitalization status, and psychological support, were not significantly associated with the BDI-II score.

## Discussion

Cancer patients receiving treatment at NNUH visit outpatient oncology clinics or are admitted as inpatients for various purposes, including diagnosis, chemotherapy sessions, autologous bone marrow transplants, and managing side effects or complications of treatment. These services cater to patients with solid tumors and hematologic malignancies such as leukemia, lymphomas, and multiple myeloma. Some oncological conditions, such as neutropenic fever, require referral to the emergency room followed by admission to the hospital.

In our research group, we observed an equal distribution between males and females, approximately 1:1, mirroring the broader pattern of cancer cases in Palestine. According to data from the Palestinian Ministry of Health in 2020^17^, nearly half of all cancer patients were male (49.3%), with slightly more females (50.7%). Conversely, a study conducted in Italy demonstrated a greater proportion of female participants, accounting for 58% of the sample^18^.

In our research, the average age of the individuals involved was 47 years, whereas in previous studies, the average ages ranged from 49.12 years^19^ to 61.9 years^18^. In our study, 88.9% of the individuals diagnosed with cancer received treatment, while the remainder were in the diagnostic phase. This proportion closely resembles that found in an earlier study, which also focused on patients undergoing chemotherapy, where 82% were in the treatment stage^18^.

In Palestine, depression represents approximately 15.3% of mental disorders^20^. In this study, 38.4% of cancer patients had minimal depression, 22.5% had mild depression, 22.1% had moderate depression, and 17.0% had severe depression based on the BDI-II scale. On the other hand, a study conducted in Gaza Strip, Palestine, used the same scale (BDI) and reported that 7.7% of cancer patients were minimally depressed, 15% were mildly depressed, 53.4% were moderately depressed, and 24.2% were severely depressed. Another study used a different scale (the center for epidemiological studies depression scale, CES-D) and reported that 44% of Palestinian cancer patients had severe depression^21^. In another population group in Palestine, 33.9% of hemodialysis patients were moderately depressed, and 29% had severe depression^22^.

The elevated rate of depression in Palestine could be linked to major stress factors such as the enduring siege and occupation^23^, heightened anxiety levels^24^, and challenges in obtaining healthcare services^25^.

In comparison to research conducted in other regions, various studies have examined the prevalence of depression among breast cancer patients. In Jordan, 52.7% of respondents experienced minimal depression, 26.0% displayed mild symptoms, 19.5% exhibited moderate symptoms, and 1.8% had severe symptoms^19^. Similarly, in Turkey, 52.0% of breast cancer patients scored 17 or higher on the BDI scale^26^. Another study employed the hospital anxiety depression scale (HADS) to assess depression among cancer patients, revealing prevalence rates of 23.1% for mild depression, 11.1% for moderate depression, and 2.3% for severe depression^27^. A cross-sectional investigation in Milan, Italy, utilizing the HADS reported that 4.1% of cancer patients experienced severe depression^18^. Moreover, a study in Greece using the Greek translation of the BDI-21 revealed that 69.5% of respondents scored above 10 (indicating mild depression), 39% scored above 16 (indicating moderate to severe depression), and 11.4% scored above 30 (indicating severe depression). Notably, women were more prone to depression than men, with a significant portion experiencing mild to severe depression^28^. Unfortunately, there is a lack of specialized centers in Palestine that are crucial for mitigating depression symptoms in cancer patients^29^.

In the current study, we found that depression was more prevalent in cancer patients aged > 50 years. As previously demonstrated in a study using the HADS for breast cancer patients undergoing radiation therapy in Palestine, age older than 51 years was associated with a greater risk of depression^30^. A cross-sectional study conducted in two Jordanian hospitals concluded that age was not significantly associated with BDI-II scores^19^.

Regarding educational level, we found that cancer patients with low educational levels had more depression, which is similar to the findings of other studies^31,32^. However, other publications did not show a significant difference between the two variables of education level and depression^19,33^. Depression was also associated with socioeconomic status since patients with poor socioeconomic status had more depression. However, compared to other studies, depression was not significantly associated with socioeconomic status^19,32^.

Our findings revealed that smoking cancer patients had greater depression scores than nonsmokers. Smoking habits may develop through stressful life events. For example, in Palestine, many people complain of psychological problems resulting from traumatic events from the Israeli occupation^20,24,34^ and anxiety^24,25^. Smokers account for more than one-fifth of people aged 18 and over; according to the Palestinian Household Survey conducted by the Palestinian Central Bureau of Statistics (PCBS) in 2010, 22.5% of Palestinians aged 18 and over in the Palestinian territory are smokers (26.7% in the West Bank compared to 14.6% in the Gaza Strip). The Jenin Governorate had the highest percentage of smokers (32.2%), while the North Gaza Governorate had the lowest percentage (11.3%)^35^. Smoking is also common in cancer patients^36^. Additionally, other studies have corroborated reports of diminished well-being among smokers^37,38^. The findings of the current study revealed that psychosocial assistance was not associated with lower depression scores; however, these findings contradict previous research that suggested that cancer patients should take advantage of accessible psychological support services to reduce their depression and that cancer patients should take advantage of available psychological support services to reduce their depression^39,40^.

Additionally, the analysis of depression levels revealed a significant link between socioeconomic status and smoking habits, consistent with prior research^41–44^. Furthermore, there was a correlation between anxiety levels and educational attainment among cancer patients. Those with lower education levels reported higher anxiety scores, while those with higher education levels showed lower scores, indicating a potential protective effect of higher education on long-term anxiety and sadness^45^. Our study also revealed that anxiety levels were greater in cancer patients at the diagnosis stage than in those undergoing treatment, in line with previous research that identified chronic inflammatory conditions as risk factors for anxiety and depression in cancer patients, particularly during the diagnostic phase^46^.

The 4.8% prevalence of deformities reported in our study encompasses various types of deformities, including tal Hashomer syndrome. However, due to the rarity of Tal Hashomer syndrome, its specific prevalence within our study population is also very low. The literature has noted infrequent instances of Tel Hashomer syndrome and Guillain‒Barré syndrome among lymphoma patients^47^.

The scales utilized in our research possess several advantages. First, by assessing ten symptoms, they allow for the identification of symptom patterns and enable swift evaluation. Moreover, these scales are widely adopted by clinical and research institutions globally, underscoring their broad acceptance. Their validity has been confirmed through psychometric testing, and they are accessible in more than 20 languages, making them usable across diverse populations. Furthermore, they exhibit minimal clinically significant differences and demonstrate high sensitivity to changes over time. Finally, these scales are freely accessible, facilitating their utilization in both clinical and research contexts^48^.

The strengths of the BDI-II are that it is user-friendly, applicable across international age groups (13 years and older), has a low reading level (average Flesch‒Kincaid grade level 3.6), and provides a substantial foundation for further research^49^.

### Strengths and limitations

This study involved cancer patients from across Palestine, encompassing both the West Bank and the Gaza Strip, representing diverse socioeconomic backgrounds. This is an inaugural Palestinian investigation shedding light on the occurrence of depression symptoms among cancer patients. Nonetheless, this study has several limitations. Among these limitations is its cross-sectional design, which inhibits the examination of how depression in cancer patients progresses with varying treatment modalities. Further limitations include the use of convenience sampling solely from a single tertiary hospital, a restricted sample size, and a predominantly hematologic malignancy-focused sample, potentially skewing the representation of broader cancer demographics. Consequently, the findings may lack generalizability.

## Conclusions

This study highlights the high prevalence of depression symptoms among cancer patients. The beck depression inventory-II (BDI-II) score was associated with factors such as age, educational level, socioeconomic status, and smoking, underlining the complexity of addressing depression in this population. Our findings underscore the utility of the BDI-II as a tool for assessing and managing depression symptoms in cancer patients, complementing the broader spectrum of care that includes psycho-oncological and psychiatric support. This integrated approach is crucial for enhancing the quality of life of cancer patients throughout their disease trajectory.

### Recommendations

- Depression symptoms were reported and referred to a social worker or available psychosocial personnel.
- Involve a psychiatric/mental health nurse or social worker in the session to break the bad news about the cancer diagnosis.
- More research is recommended considering different hospitals in Palestine and sample randomization.

## Data Availability

The data from our surveillance are not publicly available due to privacy and ethical restrictions. However, individuals interested in using the data for scientific purposes can request permission from the corresponding authors. Those with granted access will receive anonymized data to maintain patient privacy and data integrity. This manuscript is part of the Master of Community Mental Health Nursing graduation project submitted to An-Najah National University. It has been published as part of self-archiving in institutional repositories, accessible through the university repository (https://repository.najah.edu/server/api/core/bitstreams/de047bba-0136-4a7b-9e9d-a484ad5126d1/content).

## Abbreviations

BDS: Beck depression scale
BDI II: Beck depression inventory
NNUH: An-Najah National University Hospital
QoL: Quality of life
AutoBMT: Autologous bone marrow transplant
MOH: Ministry of Health
USA: United States of America
PCBS: The Palestinian Central Bureau of Statistics
CES-D: Center for epidemiological studies depression scale
HADS: Hospital anxiety depression scale
